# Supplementary material for: LRH-1 mitigates intestinal inflammatory disease by maintaining epithelial homeostasis and cell survival
Source: Nat Commun. 2018 Oct 10;9:4055. doi: 10.1038/s41467-018-06137-w (PMC6180039; doi:10.1038/s41467-018-06137-w)
Supplement: Supplementary file 1 — Supplementary Information [file 41467_2018_6137_MOESM1_ESM.docx]

**
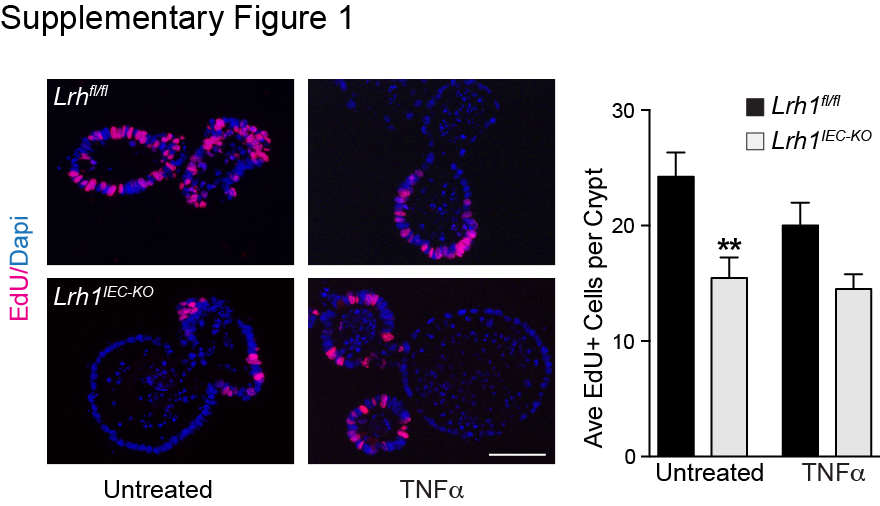
**

**Supplementary Figure 1. Loss of LRH-1 reduces overall cell proliferation in enteroids.** Proliferating cells are marked by Edu incorporation (pink nuclei). Treatment with 10 ng/ml of TNFα does not significantly depress proliferation further (middle panel). Data are quantitated at right by counting number of EdU^+^ nuclei per crypt over 20 consecutive crypts. Scale bar = 50 μm. Error bars are SEM with statistical analyses determined by Student unpaired t-test, 2-tailed with p values ** p = < 0.001.

**
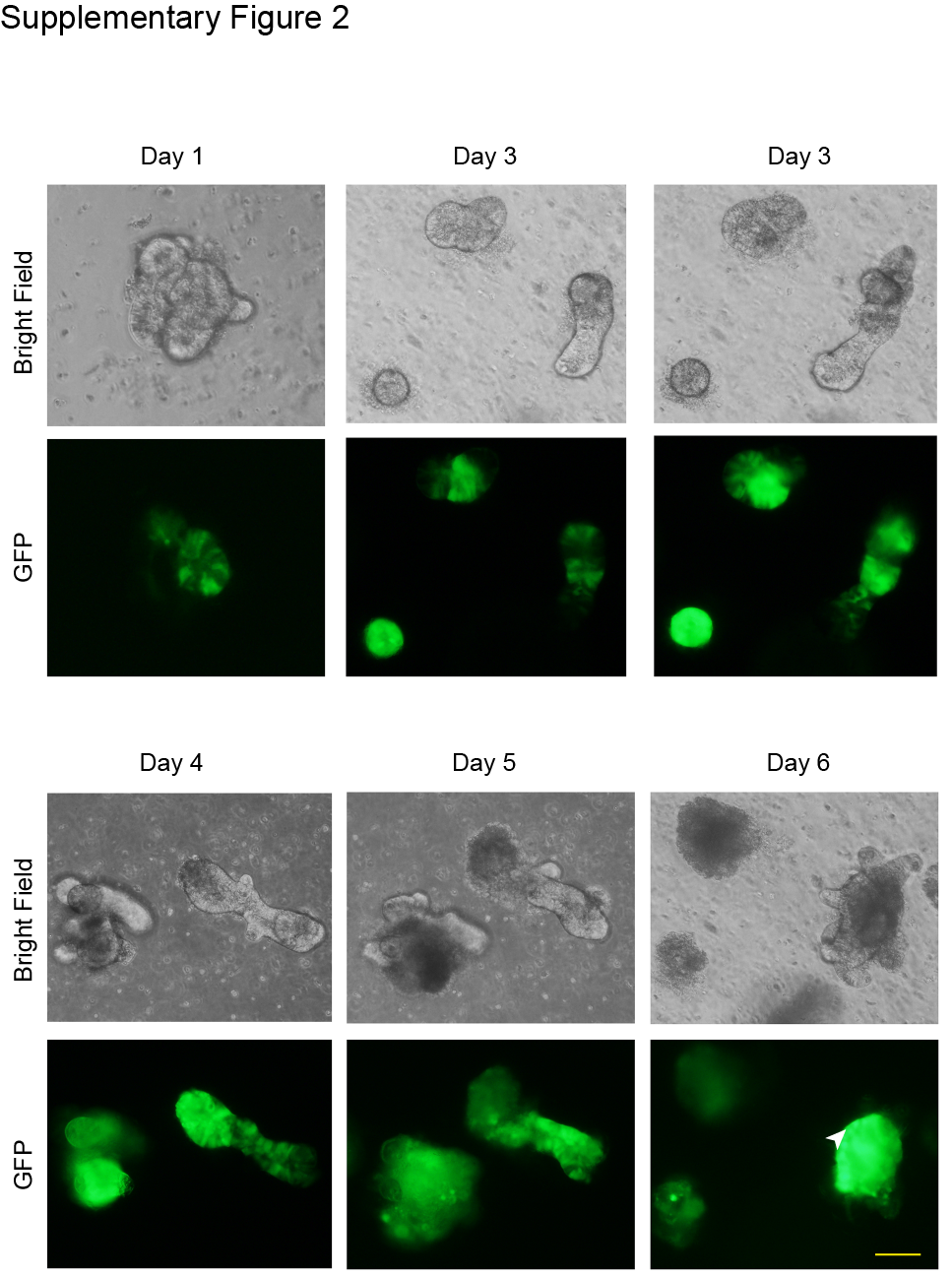
**

**Supplementary Figure 2. Time course of AAV-directed GFP expression.** GFP is detected on Day 1 post-infection. The signal reached maximal expression by Days 4-5. By Day 6 the infected cells are mainly shed in the enteroid lumen as part of normal cell turn-over. Scale bar = 100 μm.

**
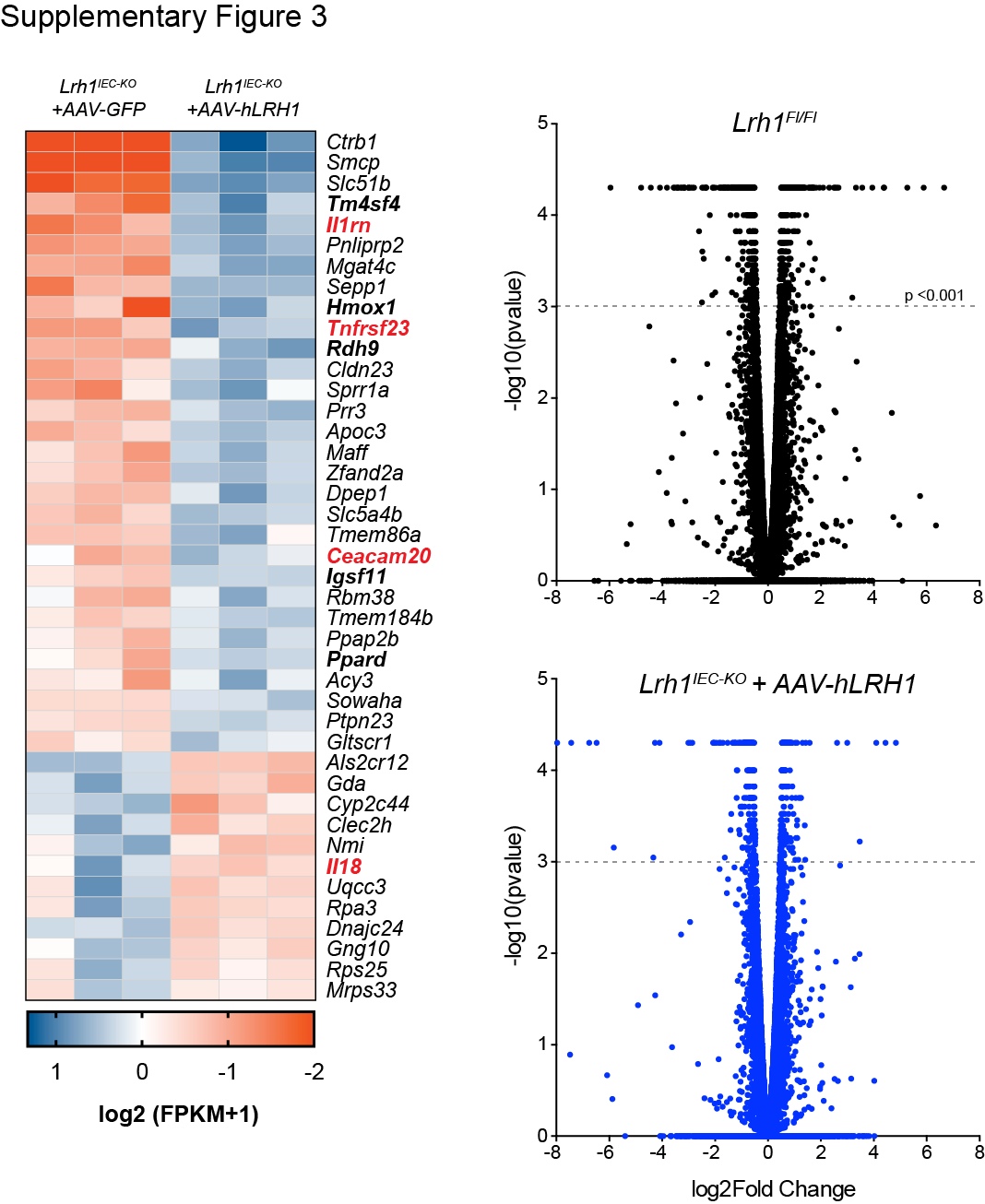
**

**Supplementary Figure 3. Expression analysis of *Lrh1^IECKO^*+AAV-hLRH1 reveals pro-survival and anti-inflammatory gene program.** Expression heat map of top 40 most significant expression changes comparing *Lrh1^IECKO^*+AAV-hLRH1 to *Lrh1^IECKO^*+AAV-GFP. Putative pro-survival and anti-inflammatory genes are highlighted in black and red, respectively. Volcano plots for *Lrh1^fl/fl^* (top right) and *Lrh1^IEC-KO^+AAV-hLRH1* (bottom right) show similar distribution and fold change. P < 0.001 is marked by dashed grey line.

**
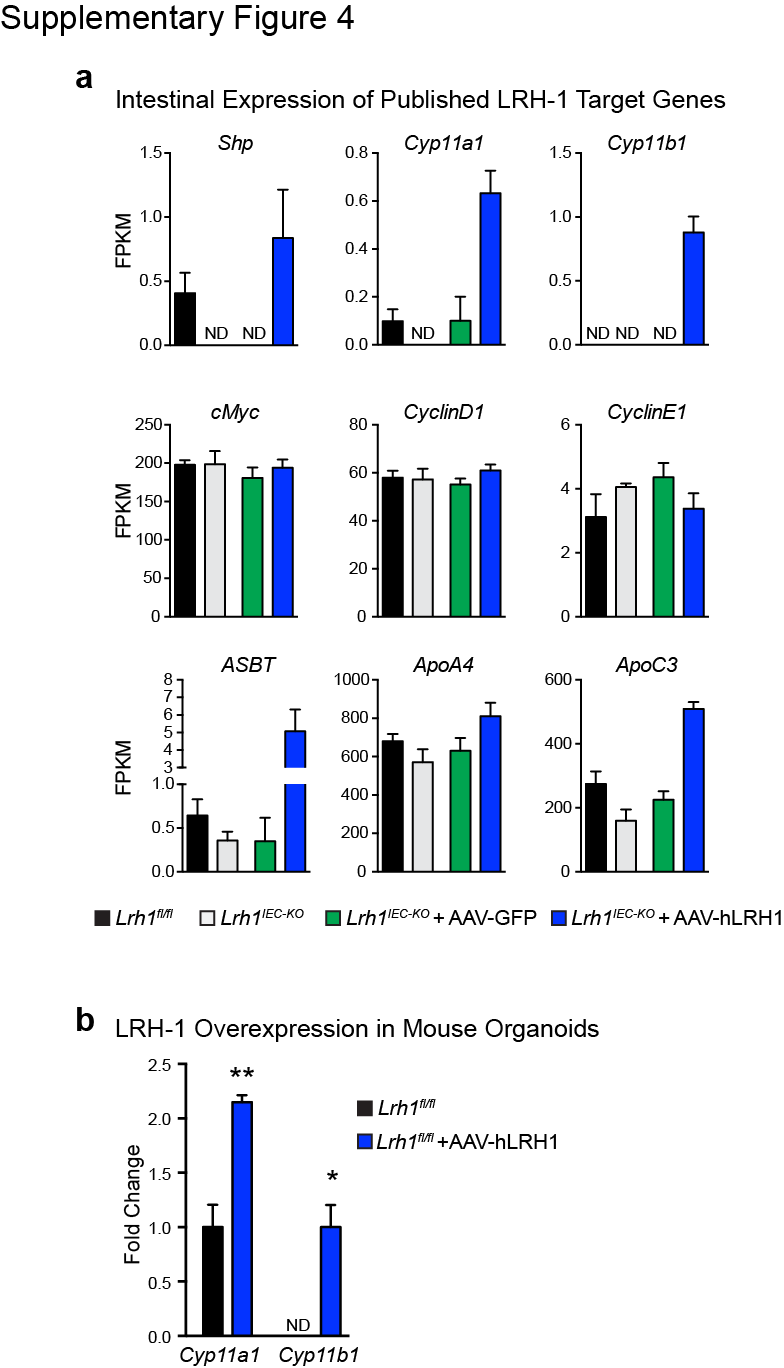
**

**Supplementary Figure 4. Effect of LRH-1 knock, replacement, and overexpression of previously recognized gene targets. (A)** Expression of target gene mRNA from RNA-Seq dataset. No change in *Myc* or *Cyclin* expression was observed following *Lrh1* knockout. **(B)** Overexpression of hLRH-1 in mouse *Lrh1^fl/fl^* organoids increases expression of the steroidogenic enzymes *Cyp11a1* and *Cyp11b1*.

**
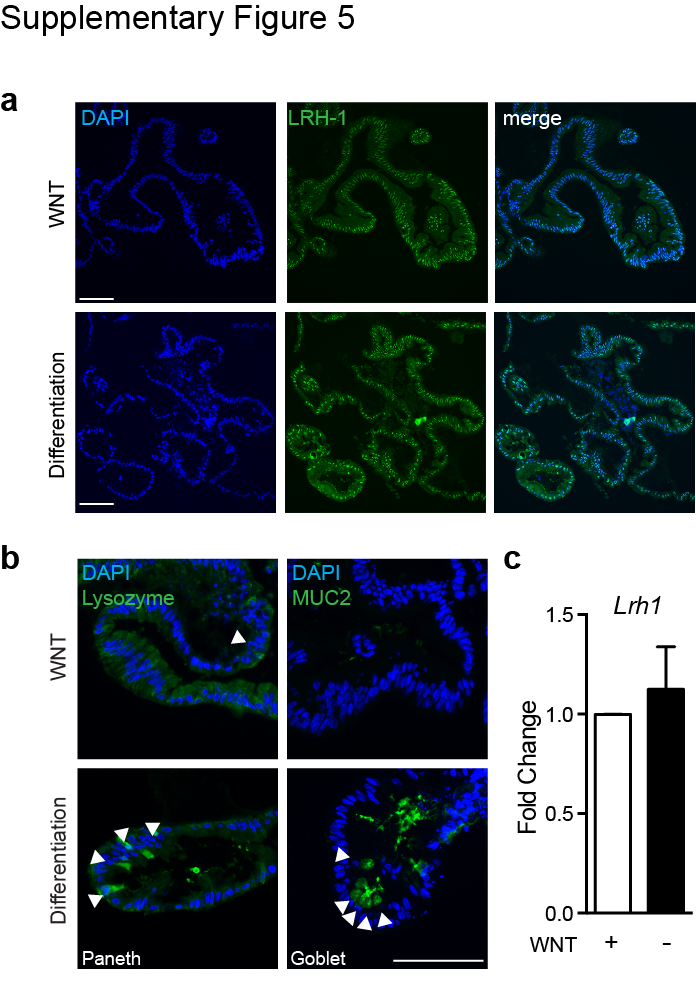
**

**Supplementary Figure 5. Differentiation and LRH-1 expression in human intestinal organoids.**

**(A)** Distribution of LRH-1 expression (green) in undifferentiated (top row) and differentiated human intestinal organoids. In both conditions, LRH-1 is expressed broadly, with mild reduction in staining intensity in differentiated villus domain. Differentiation is induced by withdraw of WNT, SB202190, and nicotinamide for 5 days. **(B)** Differentiation conditions produce both mature Paneth cells (left) marked by lysozyme expression (green; white arrowheads) and goblet cells (right row) marked by MUC-2 expression (green; white arrowheads). **(C)** *Lrh-1* mRNA levels are similar under WNT (white bar) and differentiation (black bar) conditions. Scale bars = 100 μm.

**
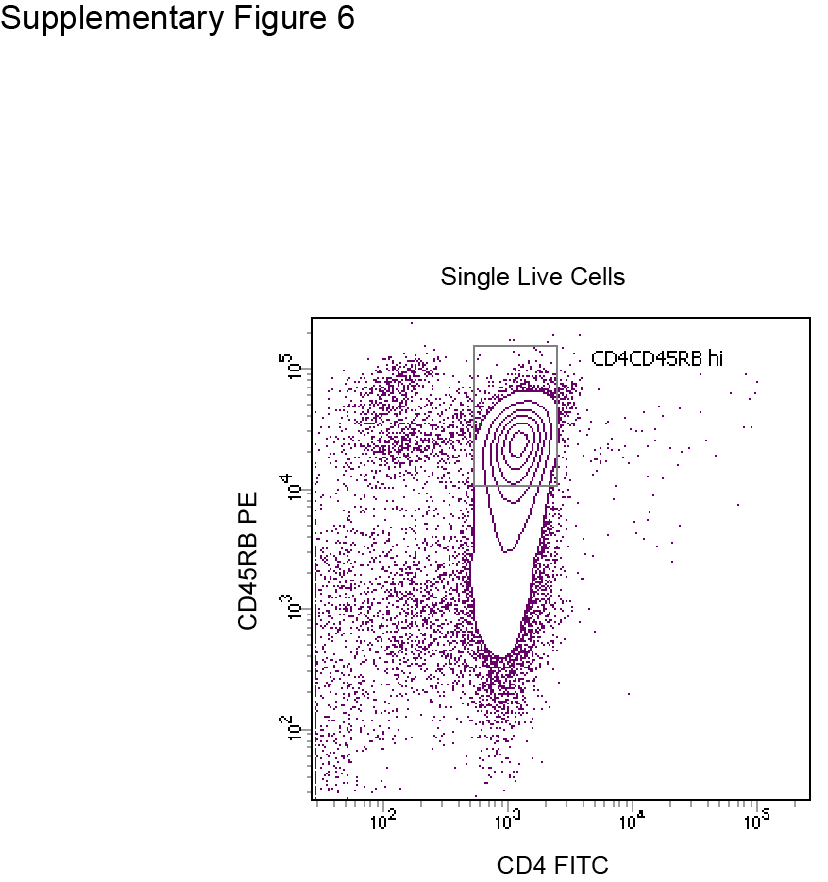
**

**Supplementary Figure 6. Gating strategy for FACS of CD4+ T cells.** Single live CD4^+^ T cells were gated on their fluorescent intensity and percentage to obtain finally CD4^+^CD45RB^high^ naïve T cells. x axis, anti-CD4 FITC intensity; y axis, anti-CD45RB PE intensity; Percentage (%) equals the percent of CD4^+^CD45RB^high^ T cells (the squared area) in the CD4+ single live cells.
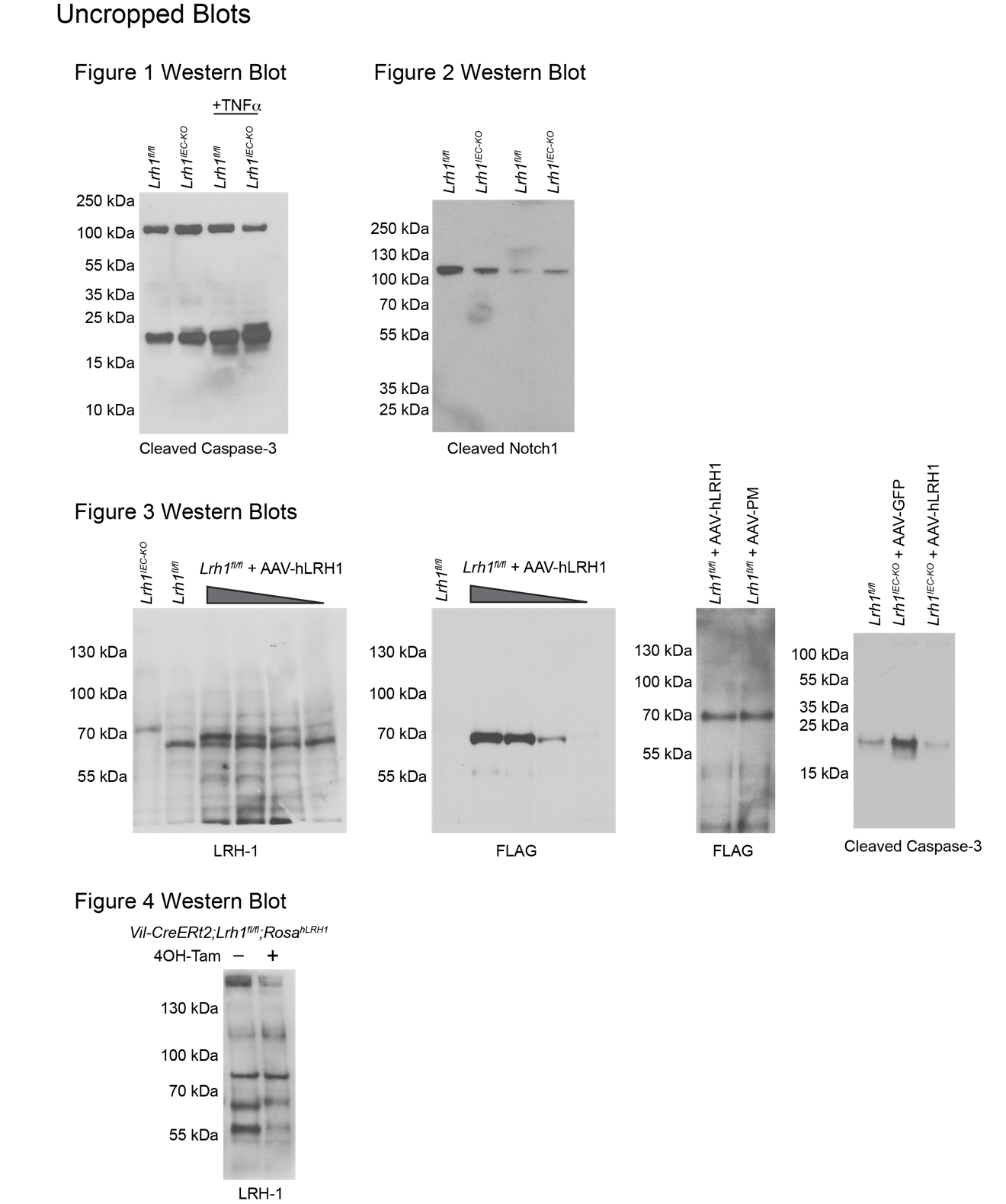


**Supplementary Figure 7. Uncropped western blots from main text figures.** Antibodies used are listed below the blot with dilutions as per Methods.

**Supplementary Table 1. Modified Colitis Disease Activity Score**

| Scores | 0 | 1 | 2 | 3 | 4 |
| --- | --- | --- | --- | --- | --- |
| Weight loss (%) | 0 | >0% | ≥2% | ≥4% | ≥8% |
| Diarrhea | Normal | Mild | Loose | Moderate | Liquid |
| Hematochezia | No | FOBT± | FOBT+ | FOBT++ | FOBT+++ |
| Sick appearance | Normal | Mild  uncleanness | Moderate  uncleanness | Hunched,  Slow moving | Lethargic |

**Supplementary Table 2. qPCR Primers**

| **Gene** | **Forward (5’**→**3’)** | **Reverse (5’**→**3’)** |
| --- | --- | --- |
| *Ctrb1(m)* | CCTGAAAATCGCTCAGGTTTT | CATTACGCACGGTGAAGGA |
| *Ctrb1(h)* | GGAGTTTGACCAGGGCTC | AGCAGGGTGATGTCATTGTTC |
| *Cyp11b1* | TGCCCTTGGAATCCTGGATAGT | CCATTCTGGCCCATTTAGCAA |
| *Hmox1* | AGGATTTGTCTGAGGCCTTG | GGCCGTGTAGATATGGTACAAG |
| *Il1rn* | TTAGCTCACCCATGGCTTCAG | GCATCTTGCAGGGTCTTTTCC |
| *Lrh1(h)* | AAGCGTTGTCCTTACTGTCG | CCCTGTCTCTCTTGTACATTGG |
| *Lrh1(m)* | CCATTACGGTCTCCTCACGTG | AGGGACATCGTTTTCTCTGCG |
| *Rdh9* | CCAGACCAGCTCAGAAATCAG | CTCTTGTCCAATTCGTTTAGCC |
| *Smcp* | CGAGAATCAAGTATGGAAATGCTG | GGGCAACATGGTTTTGGTG |
| *Tnfrsf23* | CCAGTCTAATGATGTCTGTTGCAA | TGCAGGGCGCCTTGA |

Proprietary, pre-validated primers for qPCR for colonic gene expression were purchased from Qiagen (QIAGEN Inc., Germantown, MD 20874) (<https://www.qiagen.com/geneglobe/default.aspx)>.
